# Proof of concept for low dose and delayed release of tigilanol tiglate in the treatment of equine sarcoids

**DOI:** 10.3389/fvets.2026.1807796

**Published:** 2026-08-28

**Authors:** Gideon P. Stemmet, Peter Parsons, Jenny Johns, Paul Reddell, Raphael Labens

**Affiliations:** 1 School of Animal and Veterinary Science, Faculty of Science, Charles Sturt University, Wagga Wagga, NSW, Australia; 2 QIMR Berghofer Medical Research Institute, Hertson, QLD, Australia; 3 QBiotics Group Ltd., Yungabarra, QLD, Australia

**Keywords:** beads, calcium sulphate, cutaneous neoplasia, elution, metronomic

## Abstract

Intra-tumoural administration of tigilanol tiglate has been shown effective in local treatment of cutaneous equine neoplasias. The drug causes rapid oncolysis and tumour slough, but may also be associated with comorbidities in sensitive and/or intricate anatomical sites due to its initial pro-inflammatory mode of action. Here we describe two approaches to optimise and further de-risk treatment of equine sarcoids with tigilanol tiglate, especially in difficult locations. Firstly, we provide preliminary clinical and observational data on the efficacy of low dose administration of tigilanol tiglate (metronomic or single intra-tumoural administration). Secondly, we provide proof of therapeutic concept for tigilanol tiglate eluting calcium sulphate beads by evaluating elution characteristics and assessing clinical efficacy treating equine sarcoids. Clinical efficacy, at substantially lower than previously reported tigilanol tiglate treatment doses, was demonstrated by adopting either a single low-dose or metronomic dosing schedule or an eluting implant. Treatments resulted in complete tumour slough in all instances while evoking a perceived reduction in inflammatory responses compared to the use of previously established higher doses for equine sarcoids. While implanted calcium sulphate beads loaded with cytostatic drugs have been used previously for local treatment of equine neoplasia, their utility for local delivery of tigilanol tiglate has not been explored before. Elution analysis of tigilanol tiglate from 5 and 2.5 mm loaded calcium sulphate beads showed beads eluted ~40% and ~24% of their tigilanol tiglate content in horse serum (HS) with continued release observed from intact beads for at least 4 days. The ease of implanting absorbable tigilanol tiglate loaded calcium sulphate beads, and its clinical efficacy in sarcoid treatment, showed the potential of this approach to eliminate common challenges associated with intra-tumoural injection such as inadvertent diffusion from injection sites or the difficulty injecting fibrous tumours. This contributed to reducing the apparent extent of local inflammatory responses and limiting possible associated morbidities. Apart from mild leukotrichia that developed at some treatment sites, healing and cosmetic outcome was deemed excellent. Ongoing studies evaluating the clinical efficacy and safety of lowered tigilanol tiglate doses in a statistically relevant, representative equine population are required to further develop the reported treatment regimens.

## 1 Introduction

Intra-tumoural administration of tigilanol tiglate (a diterpene ester, epoxytigliane class) has demonstrated therapeutic efficacy in treating cutaneous neoplastic masses in various species including horses, causing rapid oncolysis and tumour slough (1–10). However, aspects of its therapeutic mode of action associated with disruption of local vasculature, up-regulation of pro-inflammatory cyto- and chemokines, and induction of a localised innate immune response are also associated with potential comorbidities. This is particularly the case in sensitive and/or intricate anatomical sites and may require pre-emptive management considerations to control ensuing tissue responses and diffusion of therapeutic injections beyond primary target areas (2). For example, exceeding a single therapeutic dose of 1.5 mg of tigilanol tiglate (equivalent to 1.5 mL of Stelfonta®) in equine periocular sarcoids has been associated with a greater risk of complications given the level of ensuing inflammation and impact on ocular function (1, 2).

Metronomic chemotherapy, an alternative tumour treatment approach that involves frequent and regular administration of conventional chemotherapeutic agents at lowered doses, has been applied in veterinary and human patients to improve treatment and reduce adverse effects (11, 12). Consequently, delivering tigilanol tiglate in slow release formulations or in metronomic schedules (i.e., lower but repeated doses) could prove beneficial in controlling side effects associated with the drug’s mode of action (2).

Similarly, given the often dense tissue consistency of cutaneous tumours and associated difficulty to inject intra-tumourally, the potential for optimising dose delivery using drug-eluting implants carries additional merit. Calcium sulphate has distinct surface properties that make it an ideal drug carrier (13–15). Its ability to bind to neutral, positive and negatively charged molecules allows it to bind a diverse range of drugs (13–15). Biodegradable, biocompatible and widely available, calcium sulphate has proved to be a cost-effective carrier that can be tailored into absorbable beads of various shapes and sizes, loaded with appropriate drugs for the relevant purpose (15–18). As calcium sulphate rapidly dissolve, it allows a sustained and controlled release of loaded drugs over time (19). Elution characteristics of multiple antimicrobial and cytostatic drugs from calcium sulphate beads have been established supporting their use for treatment of local infections and neoplasia (18–22). Their utility for local delivery of tigilanol tiglate has so far not been explored.

With the overarching aim to optimise and further de-risk treatment of equine sarcoids with tigilanol tiglate, our first objective was to provide preliminary clinical and observational data on the efficacy of low dose administration of tigilanol tiglate (metronomic or single intra-tumoural administration) at 0.05 to 0.1 mg/cm^3^ of tumour volume. Our second objective was to provide proof of therapeutic concept for tigilanol tiglate eluting calcium sulphate beads by first evaluating elution characteristics and then clinical efficacy when treating equine sarcoids. We hypothesised that: (1) low dose administration of tigilanol tiglate (metronomic or single intra-tumoural administration) would achieve complete tumour resolution while limiting local tissue responses and comorbidities; (2) elution rate of tigilanol tiglate from larger (5 mm) beads would be greater compared to smaller (2.5 mm) beads; (3) tigilanol tiglate released from loaded beads would maintain sufficient bioactivity to achieve complete tumour resolution in treated sarcoids.

## 2 Methods

### 2.1 Population/horses

Six horses with skin tumours were presented to the Veterinary Clinical Centre and underwent experimental treatment with institutional animal care and ethics committee approval numbers: A24375 and A21394. Owners were informed of conventional treatment options first before giving signed consent to receive experimental treatment for which costs were fully supported by the study sponsor (QBiotics Group Ltd). Signalment and tumour descriptions were recorded at initial assessment and are summarised below (Table 1). Preceding treatment, physical examinations were performed to ascertain the horses’ general health status and screen for abnormal vital signs that would preclude them from trial inclusion.

**Table 1:** Signalment and tumour descriptions.

Horse	1. 6-year-old ASH gelding		2. 4-year-old AND mare	3. 6-year-old WB gelding	4. 3-year-old ASH gelding	5. 10-year-old TB gelding	6. 5-year-old Arab mare
Tumour ID	T_I_	T_II_	T_III_	T_IV_	T_V_	T_VI_	T_VII_
Tumour site	Lateral canthus of the right eye and adjacent upper and lower eyelids	Proximal dorsal aspect of the left-fore antebrachium	Medial canthus of the right eye and dorsal eyelid	Right dorsal peri-ocular (dorsal orbital rim)	Apex nasi, dorsal rostral region between the nostrils	Right medial/cranial pinna	Right ventral muzzle
Tumour description	Fibroblastic	Fibroblastic	Mixed -verrucose/ fibroblastic	Mixed- verrucose/ occult	Mixed-verrucose/ fibroblastic	Fibroblastic	Verrucose
Initial tumour measurements	2.5x2x2cm	3.3x4x1cm	5x2x0.8 cm	3x2x0.8 cm	3×1.5x1cm	A, 2.5×1.8×2.2 cm B. 2.7x2x1cm	3x2x0.8 cm
Initial estimated tumour volume	5cm^3^	6.6cm^3^	4cm^3^	9.6cm^3^	2.25cm^3^	A, 4.95cm^3^ B. 2.7cm^3^	2.29cm^3^
Treatment regimen	Repeated low dose intratumoural injection	Repeated low dose intratumoural injection	Repeated low dose intratumoural injection	Repeated low dose intratumoural injection	Single low dose intratumoural injection	Calcium sulphate loaded beads	Calcium sulphate loaded beads

AND, Andalusian; WB, Warmblood; ASH, Australian Stock Horse; TB, Thoroughbred; Arab, Arabian.

### 2.2 Tumour types and treatment regimens

Seven equine sarcoid lesions in sensitive and/ or intricate anatomical sites underwent intra-tumoural treatment with tigilanol tiglate. One horse (horse 1), presented with two sarcoid lesions (T_I_ and T_II_). Tumour diagnoses and characterisation were made based on clinical history, tumour localisation, morphology and progression (23–28). In one lesion (T_IV_) tumour characterisation was equivocal and a diagnosis was confirmed by histopathological assessment. Treatment regimens were assigned sequentially to newly presenting clinical cases on a response-guided approach based on the observed clinical response of the previous intervention.

Five lesions underwent low dose metronomic intra-tumoural injections, before the response to a single low dose treatment was evaluated in one case. With the support of observed treatment responses, the therapeutic effect of tigilanol tiglate eluting implants was subsequently assessed in two clinical cases (Table 1).

#### 2.2.1 Repeat intra-tumoural injection

Four sarcoids (T_I_, T_II_, T_III_ and T_IV_) were treated by repeat low dose intra-tumoural injections. Sarcoids, T_I_ and T_II_, were present in the same horse and commenced tratement concurrently. Prior to treatment, tumours and adjacent skin were prepped (4% chlorhexidine gluconate or 10% povidone-iodine antiseptic scrub followed by 70% isopropyl wipe) and the area desensitised using locoregional anaesthesia (2% NV Mepivacaine Injection)^1^ performed remote from the tumour. Intra-tumoural injection was performed using the previously described “fanning technique” with a single injection site to minimise potential leakage of the administered dose (1). Local administration of tigilanol tiglate along tumour margins and for the treatment of verrucose and occult sarcoids was performed using a 34G, 4 mm multi-needle injector (Quatron®).^2^ To facilitate an adequate injection volume, tigilanol tiglate doses were diluted in sterile physiologic saline at a maximum 1:10 ratio. Administration regimens are listed in Table 2. As an exploratory assessment of treatment associated tissue changes in perfusion and vascularity, thermographic images of T_I_ and T_II_ were obtained on D4.

**Table 2:** Repeat tigilanol tiglate intra-tumoural injection dose and administration regimens.

Protocol component	Tumour			
	T_I_	T_II_	T_III_	T_IV_
Tigilanol tiglate dose regimen	Cumulative dose of 1.77 mg equivalent to 0.35 mg/cm^3^ over 7 consecutive treatments followed by final low dose injection at D66 D0: 0.35 mg tigilanol tiglate diluted in saline to 7 mL (7% v/v) D1: 0.25 mg tigilanol tiglate diluted in saline to 4 mL (5% v/v) D2: 0.25 mg tigilanol tiglate diluted in saline to 4 mL (5% v/v) D3: 0.3 mg tigilanol tiglate diluted in saline to 5 ml (6% v/v) D4: 0.3 mg tigilanol tiglate diluted in saline to 5 mL (6% v/v) D8: 0.16 mg tigilanol tiglate diluted in saline to 3.2 ml (5% v/v) D9: 0.16 mg tigilanol tiglate diluted in saline to 3.2 mL (5% v/v) D66: 0.14 mg tigilanol tiglate diluted in saline to 1.4 mL (5% v/v)*	Cumulative dose of 1.58 mg equivalent to 0.24 mg/cm^3^ over 5 consecutive days D0: 0.6 mg tigilanol tiglate diluted in saline to 12 mL (9% v/v) D1: 0.33 mg tigilanol tiglate diluted in saline to 6 mL (5% v/v) D2: 0.33 mg tigilanol tiglate diluted in saline to 6 mL (5% v/v) D3: 0.15 mg tigilanol tiglate diluted in saline to 3.3 ml (2.3% v/v) D4: 0.15 mg tigilanol tiglate diluted in saline to 3.3 mL (2.3% v/v)	Cumulative dose of 0.56 mg equivalent to 0.14 mg/cm^3^ over 4 treatments D0: 0.24 mg tigilanol tiglate diluted in saline to 2.4 mL (6% v/v) D1: 0.24 mg tigilanol tiglate diluted in saline to in 2.4 mL saline (6% v/v) D5: Focal retreat 0.06 mg tigilanol tiglate diluted in saline to 0.6 mL (5% v/v) D12: Focal retreat 0.02 mg tigilanol tiglate diluted in saline to 0.2 mL (5% v/v)	Cumulative dose of 0.36 mg administered in 3 treatments: D0: 0.15 mg tigilanol tiglate diluted in saline to 1 mL (5% v/v) D36: 0.17 mg tigilanol tiglate diluted in saline to in 1 mL saline (7% v/v) ^§^ D67: 0.04 mg tigilanol tiglate diluted in saline to 0.6 mL (5% v/v) ^‡^
Tigilanol tiglate administration regimen	Intratumoural injection using fanning technique Delivered using Luer lock syringe with 25G 1.5″ needle Focal retreatments on D8 & D9—Quatron® needles for injection of tumour margins	Intratumoural injection using fanning technique at base of tumour Delivered using Luer lock syringe with 25G 1.5″ needle Intratumoural injection on D0 and D3—Quatron® needles Focal retreatment on D4—Quatron® needles for injection of tumour margins	Quatron® needles for injection of region without intratumoural fanning	

Tumour volume re-estimated prior to repeat treatment courses (%) calculation: *(3.5x2x0.8 cm; 2.8 cm^3^); §(3x2x0.8 cm;2.4cm^3^); ‡ (2×1.5×0.5 cm; 0.75cm^3^). 10% v/v dosing = 0.1 mg/cm^3^ of tumour volume.

#### 2.2.2 Single intra-tumoural injection

T_V_, a fibroblastic sarcoid located on the apex nasi between the nostrils, was treated by a single low dose tigilanol tiglate intratumoural injection (Table 3). Prior preparation of the treatment site for injection mirrored that described above.

**Table 3:** Single tigilanol tiglate intra-tumoural injection dose and administration regimens.

Protocol component	Tumour
	T_V_
Tigilanol tiglate dose regimen	0.05 mg/cm^3^ (0.11 mg single dose diluted in 1.2 mL saline (5%v/v))
Tigilanol tiglate administration regimen	Intratumoural injection using Quatron® needles

#### 2.2.3 Tigilanol tiglate loaded bead implantation

Following the effective treatment of five equine sarcoid lesions adopting a low dose intra-tumoural injection regimen, a further two sarcoids (T_VI_ and T_VII_) underwent treatment with absorbable tigilanol tiglate loaded calcium sulphate beads (Table 4). Beads were implanted under standing sedation and following standard surgical preparation of treatment sites as described above.

**Table 4:** Tigilanol tiglate loaded bead implantation and dose regimens.

Protocol component	Tumour	
	T_VI_	T_VII_
Beads tigilanol tiglate content	2.5 mm beads (small) - 0.15 mg tigilanol tiglate; 5 mm beads (large)- 0.41 mg tigilanol tiglate	
Bead administration regimen	D0: (tumour site A) 5× 5 mm beads (tumour site B) 3× 2.5 mm beads D21: 3× 5 mm beads^*^	D0: 1× 5 mm and 4× 2.5 mm beads
Bead dose regimen	D0: (tumour site A) 2.05 mg [0.82] equivalent to 0.41 mg/cm^3^ [0.17] (tumour site B) 0.45 mg [0.11] equivalent to 0.17 mg/cm^3^ [0.04] D21: 1.23 mg [0.30] equivalent to 0.36 mg/cm^3^[0.09]	D0: 1 mg [0.31] equivalent to 0.44 mg/cm^3^ [0.13]

Dose regimen adjusted for elution capacity in square brackets; *Tumour volume re-estimated prior to repeat dose calculation: (1.8 × 3.2× 1.2 cm; 3.46cm^3^).

The number of beads implanted was determined based on tumour volume and the expected elution capacity (see below) with the aim to approach effective dosing schedules observed in preceding cases (see details under 2.2.1 and 2.2.2). Beads were spaced uniformly and placed via 10 mm peripheral skin incisions into the tumour base or directly adjacent to the tumour mass. Skin incisions were closed using USP 2–0 glycomer 631 suture material in a cruciate pattern. Individual treatment details are listed below (Table 4).

### 2.3 Dose determination and concurrent medications

Lowered tigilanol tiglate treatment doses were guided by earlier exploratory work and calculated based on estimated tumour volume using a simplified ellipsoid formula [(*T_vol_ (cm^3^) = 1/2 (lesion length x lesion width x lesion thickness)*)] (1, 29–31). Doses ranged between 0.05 and 0.1 mg/cm^3^ of tumour mass and are summarised for each sarcoid in Tables 2–4. Prophylactic medications to manage local acute inflammatory responses were administered at the time of treatment. All treatments were administered at a single institution by two clinical investigators with prior experience treating equine sarcoids with tigilanol tiglate. Standing sedation used included detomidine (0.01 mg/kg IV; Sedator)^3^ and butorphanol (0.01 mg/kg IV; Illium Butorgesic)^4^ with additional detomidine (0.005 mg/kg) boluses administered as required. All horses received flunixin meglumine (1.1 mg/kg IV; Flunixon Injection)^5^ at the time of treatment. Dexamethasone (0.1 mg/kg IV; Illium Dexapent; see Footnote 4) was administered at the time of intra-tumoural injections and continued once daily for 3 days. Thereafter horses were continued on non-steroidal medication (phenylbutazone; 2.2 mg/kg PO, SID - BID; Equine Bute Paste; see Footnote 3) as needed.

### 2.4 Tigilanol tiglate elution characteristics from calcium sulphate beads

#### 2.4.1 Manufacture of absorbable tigilanol tiglate loaded beads

**Figure 1:**
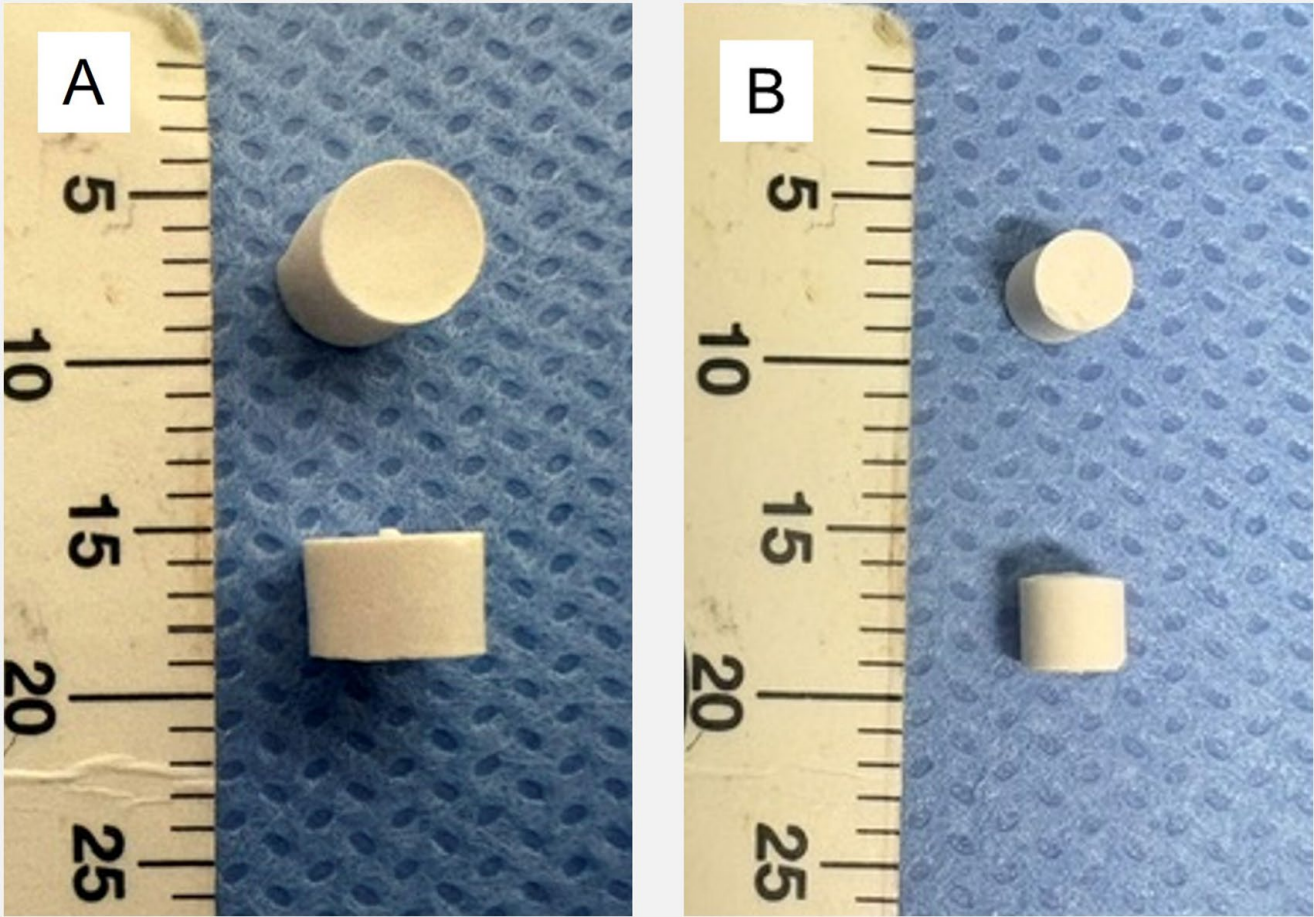
Photographs of manufactured absorbable tigilanol tiglate loaded calcium sulphate beads. **(A)** 5 mm & **(B)** 2.5 mm (top & side view).

Tigilanol tiglate powder (50 mg), calcium sulphate hemihydrate (9.5 g) from a commercially available kit (Osteoset® 5 cc resorbable mini-bead kit)^6^ and the supplied cure diluent (3 mL saline) were mixed under sterile conditions. The mixture was allowed to stand for approximately 1 min, then mixed for a further 30 s to obtain a paste and transferred to 2.5- and 5 mm silicone bead mould mats. The moulds were gently tapped on the benchtop to ensure even filling and to remove any remaining air bubbles, then left to dry at room temperature (~12 °C) for 24 h. The pellets were removed, weighed and stored aseptically at room temperature until implantation (Figure 1). Control pellets were made similarly, but without the addition of tigilanol tiglate.

#### 2.4.2 *In vitro* tigilanol tiglate loaded bead elution analysis

Three large (5 mm) beads were rocked gently while soaking in either 1.5 mL of phosphate-buffered saline (PBS) or horse serum (HS) at 37 °C for 120 h. At 0, 1, 4, 24, 48 and 120 h, 100 μL aliquots were removed without replacing elution solution, centrifuged at 1300 rpm for 20 min and filtered (Vented Millex-GV 0.22 μm)^7^ to remove particulates prior to high-performance liquid chromatography (HPLC; Shimadzu Prominence HPLC system)^8^ analysis. Three control beads were processed in a similar fashion. When PBS was used for elution, HPLC was performed using 10 μL of aliquots directly. When beads bathed in horse serum samples were extracted with ethyl acetate, twice evaporated and redissolved in ethanol prior to HPLC analysis. Following completion of elution assays (at 120 and 96 h for 5 mm and 2.5 mm beads respectively), the beads were rinsed with PBS, crushed and extracted with ethanol to determine the remaining amount of tigilanol tiglate. Small (2.5 mm) beads were analysed in a similar manner, but only one bead per elution-solution was evaluated (PBS, HS and Foetal Calf Serum, FCS) and aliquots removed at 0, 1, 4, 24, 48 and 96 h.

### 2.5 Outcome assessment/follow-up

Akin to previous reports (2), the response to treatments was assessed on the basis of periodic in person clinical examinations and sequential digital photographs provided by caretakers of treatment sites daily for the first 7 days, followed by weekly thereafter. Treatment sites were closely monitored for signs of inflammation, tumour necrosis and slough. From then on wound characteristics and healing progress was monitored as they were left to heal by second intention. Once formed, zinc oxide ointment was applied to all wounds twice daily.

Successful treatment was defined as complete tumour slough, re-epithelialisation of associated tissue deficits and treatment sites appearing likely tumour free at a minimum 10-months post treatment. Inadequate treatment responses, as judged by treating veterinarians, included no response (no change/ reduction to tumour volume), incomplete tumour slough (persistence of residual tumour tissue) or evidence of proliferative tumour growth. Any treatment that was performed outside of a regular metronomic schedule, i.e., at least 2-weeks post the initial treatment was permitted and considered a repeat treatment course.

## 3 Results

### 3.1 Repeat intratumoural injection

#### 3.1.1 T_I_—lateral canthus and adjacent upper and lower eyelids

**Figure 2:**
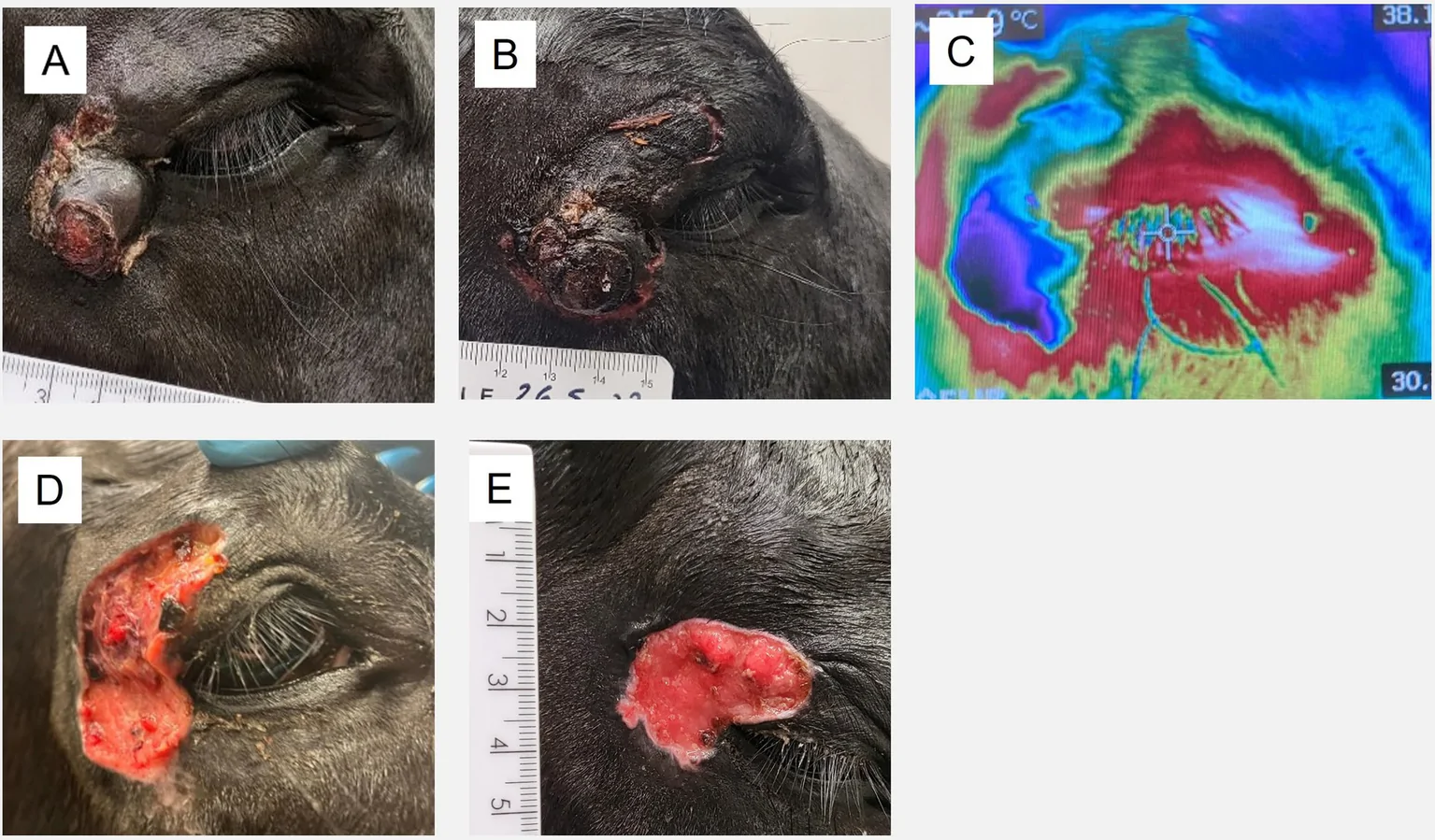
Clinical response to repeat lower dose treatments of a peri-ocular fibroblastic sarcoid (T_I_) is demonstrated. **(A)** Sarcoid prior to treatment administration (D0); **(B)** Clear margins of necrosis & exudative discharge D4; **(C)** Thermographic image showing cooling of the base of the tumour; **(D)** Complete tumour necrosis & slough (D8); **(E)** Healthy granulation tissue with evidence of re-epithelialisation & contraction at D21.

The clinical response to repeat lower dose treatments of a peri-ocular fibroblastic sarcoid is demonstrated in Figure 2. A cumulative dose of 1.77 mg was administered over seven consecutive treatments. This was followed by a final eighth administration (0.14 mg) treatment performed after 66 days. Figure 2A shows the sarcoid prior to administration of tigilanol tiglate treatment. Clear margins of necrosis and exudative discharge was noted on D4 (Figure 2B) at which time thermography confirmed cooling of the tumour relative to surrounding tissues (avascular necrosis; Figure 2C). Complete tumour necrosis and slough occurred around D8, as shown in Figure 2D. Mild peri-ocular and peritumoural swelling and oedema was present during the nine-day treatment period. Mild blepharospasm was noted in the first 24–48 h, however there was no loss of eyelid function, and no ophthalmic treatment was required. The wound deficit filled in rapidly with granulation tissue and by D21 had evidence of re-epithelialisation and contraction (Figure 2E). However, the dorsal margin maintained a slightly proliferative appearance.

**Figure 3:**
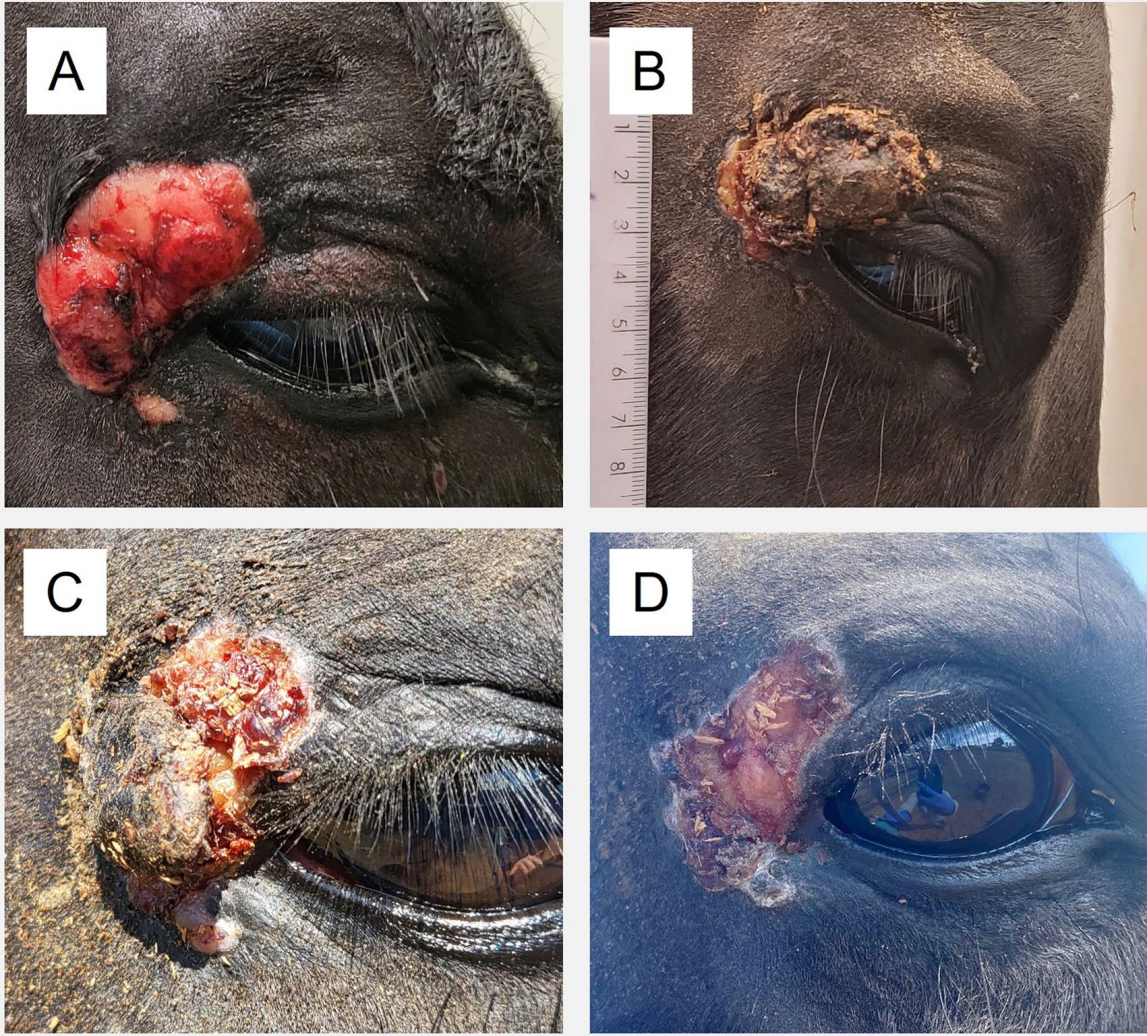
Progression of clinical response of a peri-ocular fibroblastic sarcoid (T_I_) following a repeat low dose treatment. **(A)** Failure of complete wound healing & excessive new tissue formation (treatment 2), D66; **(B)** Near complete tumour necrosis & slough D5 (treatment 2); **(C)** Eschar formation & granulation tissue D10 (treatment 2); **(D)** Wound deficit filled with granulation tissue & epithelialization D21 (treatment 2).

The horse was lost to follow-up for a period of ~5-weeks. At representation, 66 days following the first treatment, failure of complete wound healing and excessive new tissue formation was evident (Figure 3A). Assumed to represent tumour recurrence rather than excess granulation tissue formation and presented outside the allowable metronomic schedule, a repeat low dose intratumoural injection treatment was performed. At D5 (treatment 2) near complete tumour necrosis and slough was achieved (Figure 3B) with eschar formation and granulation tissue noted around D10 (T2; Figure 3C). Mild peritumoural swelling and oedema developed during the repeat treatment period. No blepharospasm or ocular discomfort was noted. The wound deficit filled with granulation tissue and epithelisation was evident at D21(treatment 2; Figure 3D) after which the horse was permanently lost to follow-up.

#### 3.1.2 T_II_—proximodorsal aspect of the left-fore antebrachium

**Figure 4:**
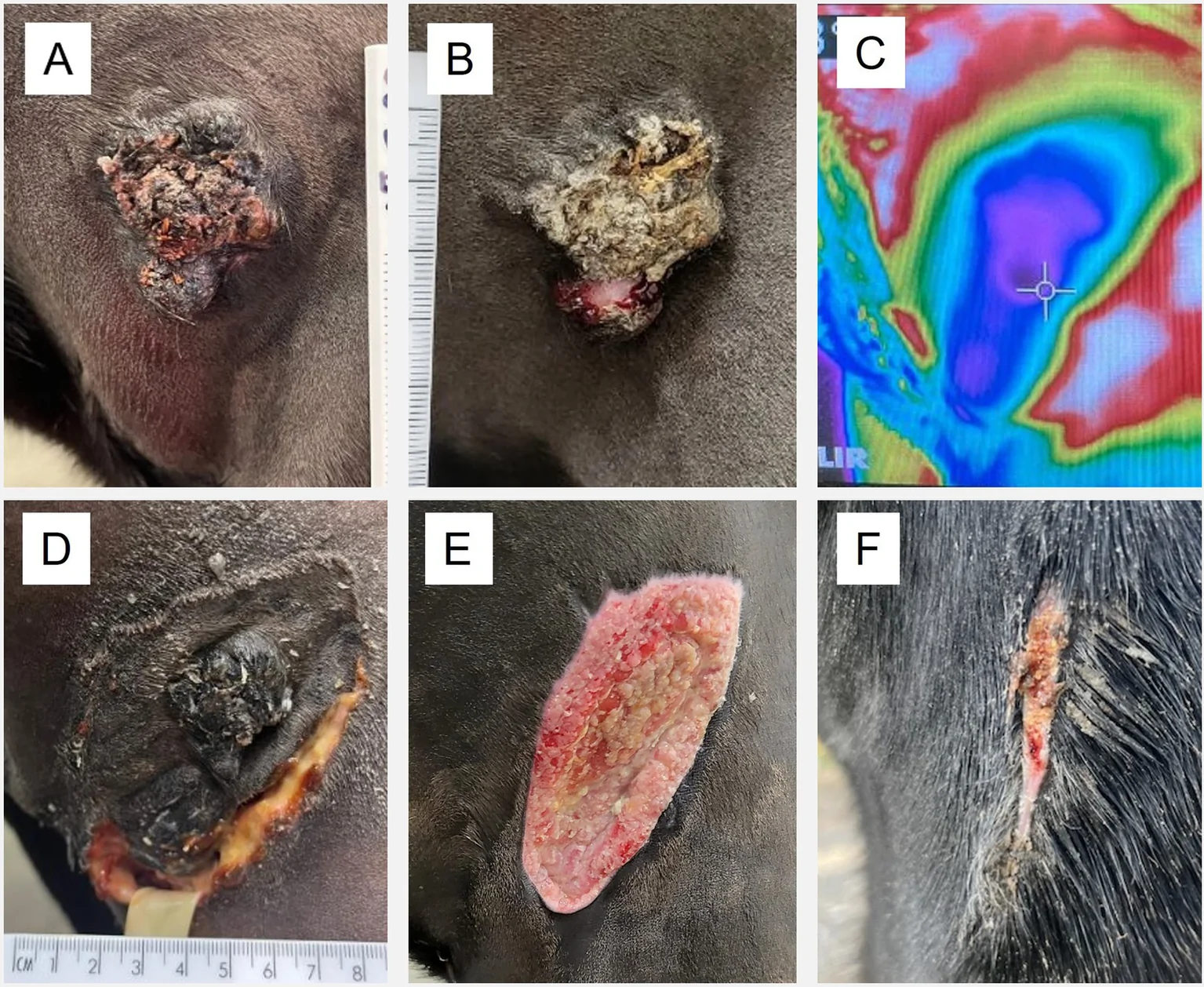
Clinical response to five consecutive lower dose administrations in a fibroblastic sarcoid of the proximal dorsal aspect of the left-fore antebrachium (T_II_) is demonstrated. **(A)** Sarcoid prior to treatment administration D0; **(B,C)** The sarcoid at D4, prior to final treatment & thermographic image illustrating cooling of the treatment site (purple area centrally and blue region extending beyond the tumour margins); **(D)** Clear margins of necrosis & moderate exudative discharge (D8); **(E)** Healthy granulation bed with wound contracture at D21; **(F)** Evidence of near complete healing D66.

The clinical response to five consecutive lower dose administrations is demonstrated in Figure 4. Figure 4A shows the sarcoid prior to administration of tigilanol tiglate treatment while Figure 4B shows the sarcoid at D4, prior to administration of the final treatment. Thermography performed at this time showed focal cooling of the treatment site, extending beyond the margins of the tumour (Figure 4C) although complete slough of the tumour had not yet occurred. Moderate peri-tumoural and distal limb swelling/oedema developed within the first 24 h following the first treatment with most significant swelling noted around day three (D3) and progressively subsiding from D5. By D8 clear margins of necrosis and moderate exudative discharge (Figure 4D) was present. A focal area of necrosis developed distal to the treatment site. Sloughing of the tumour was complete around D11. Wound healing progressed without incident with filling of the tumour wound deficit with healthy granulation tissue. At D21 a healthy granulation bed and wound contracture was evident (Figure 4E). After being lost to follow-up for 5 weeks, the horse was represented at D66 with evidence of near complete healing of the treatment site (Figure 4F). No further follow-up for this site was available thereafter.

#### 3.1.3 T_III_—medial canthus and dorsal eyelid

**Figure 5:**
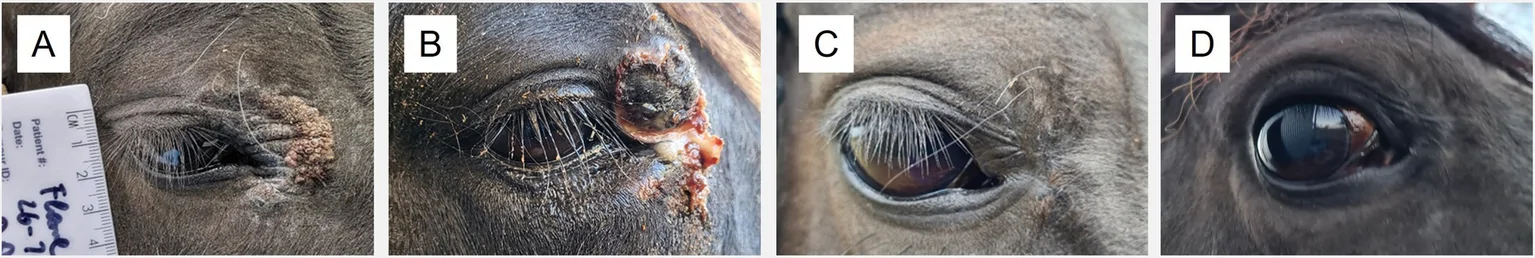
Clinical response to repeat low dose treatments of a mixed verrucose/fibroblastic sarcoid (T_III_) is demonstrated. **(A)** Sarcoid prior to treatment administration; **(B)** Clear demarcation & partial necrosis with exudative discharge (D7); **(C,D)** Complete tumour resolution at 4 and 14-months post treatment.

Clinical response to repeat low dose treatments (cumulative dose of 0.56 mg over four treatments) of a mixed (verrucose/fibroblastic) sarcoid is demonstrated in Figure 5. Although the initial response to treatment was slow with minimal swelling and mild signs of inflammation surrounding the treatment site clear demarcation and partial necrosis with mild associated exudative discharge was observed by D7 (Figure 5B). Complete tumour necrosis and slough occurred around D12 at which time a final focal retreatment along the tumour margins was administered. Healing of the wound progressed with eschar reformation around D14 and evidence of re-epithelialisation and contraction between D21 and D38. At 4-months post treatment complete tumour resolution was observed with no evidence of recurrence (Figure 5C) and /or development of sarcoids in other anatomical locations. The horse maintained a normal demeanour and appetite throughout the treatment. There was no evidence of eyelid function impairment at 14-month follow-up (Figure 5D).

#### 3.1.4 T_IV_—dorsal peri-ocular region

**Figure 6:**
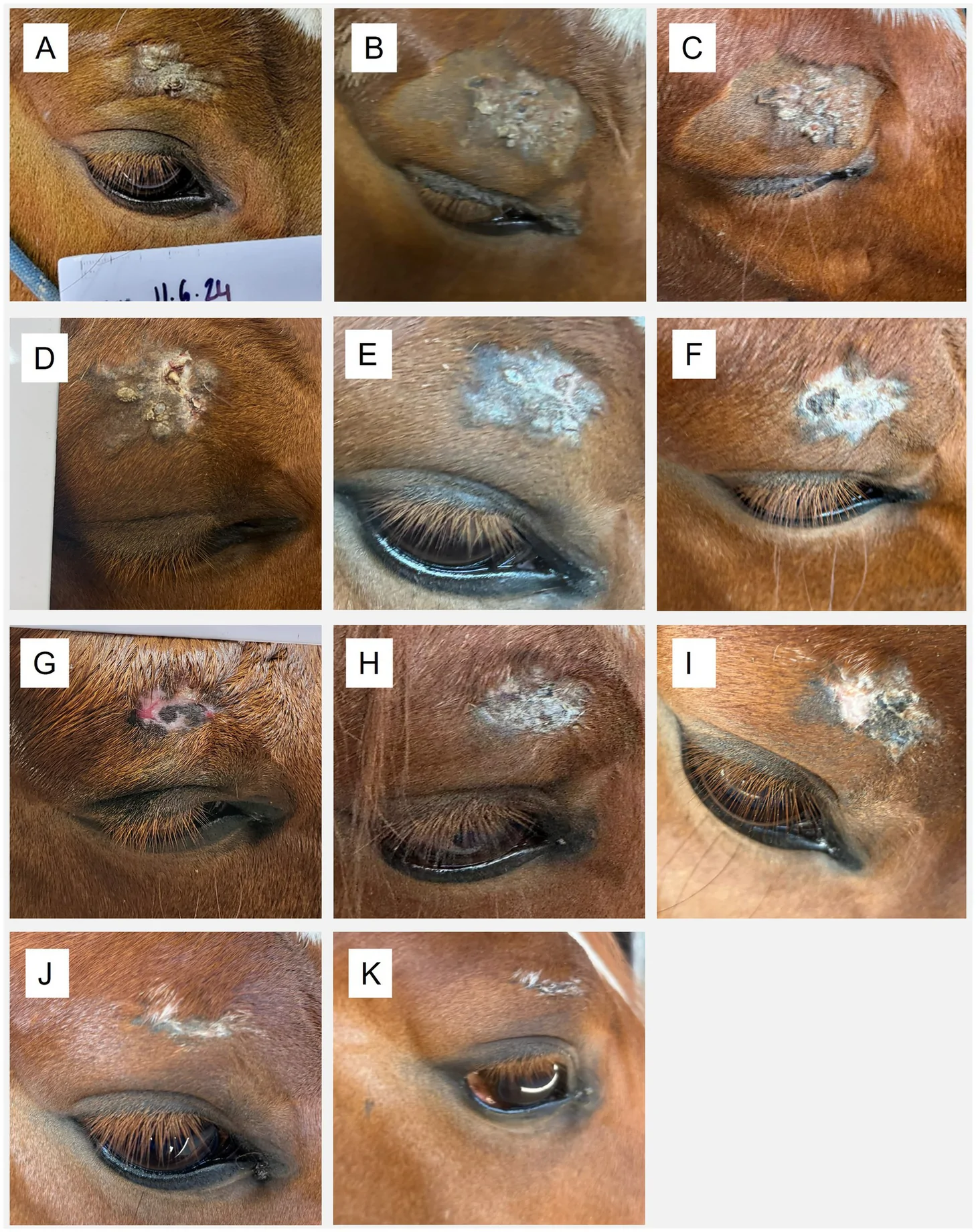
Clinical response following repeat low dose treatments of a mixed verrucose/occult sarcoid (T_IV_) is demonstrated (Horse 3). **(A)** Sarcoid prior to administration of treatment; **(B**,**C)** shows the sarcoid at D5 & D8 post first treatment (T1); **(D)** D36 (T1) presence of residual tumour; **(E**,**F)** mild exudative discharge, eschar formation & granulation; **(G)** failure of re-epithelialisation D31 (T2); **(H**–**J)** progressive healing of wound following T3; **(K)** complete tumour resolution with mild scarring & leukotrichia.

The clinical response following three repeat treatment courses with low dose intratumoural treatments (cumulative dose of 0.36 mg) of a mixed (verrucose/occult) sarcoid is demonstrated in Figure 6. Figure 6A shows the sarcoid prior to administration of tigilanol tiglate treatment while Figures 6B,C shows the sarcoid at D5 and D8 post first treatment (T1). The initial response to treatment was slow with mild signs of swelling and oedema (inflammation) surrounding the treatment site. The upper eyelid function was not altered and no signs of blepharospasm were noted. By D36 (T1) the response to treatment was deemed inadequate with minimal change in tumour volume and presence of residual tumour (Figure 6D) and a repeat low dose treatment was administered D0 (T2). This repeat treatment led to further demarcation and necrosis of the sarcoid over the ensuing 4- days with development of mild exudative discharge, eschar formation and subsequent granulation of the shallow tissue deficit (Figures 6E,F). Failure of re-epithelialisation and wound healing was evident at D31 following the second administered treatment (T2) and a third repeat low dose treatment (T3) was performed (Figure 6G). Minimal swelling and associated oedema developed during the repeat treatments using low dose tigilanol tiglate. Re-assessment at 12-months post final treatment (T3) confirmed complete tumour resolution with no evidence of recurrence at the treatment site. Mild scarring and leukotrichia at the treatment site were present with no development of additional sarcoids elsewhere during this time (Figure 6K). The horse maintained a normal demeanour and appetite and continued ridden work throughout the treatment period.

### 3.2 Single intratumoural injection

#### 3.2.1 T_V_—apex nasi, dorsal rostral region between the nostrils

**Figure 7:**
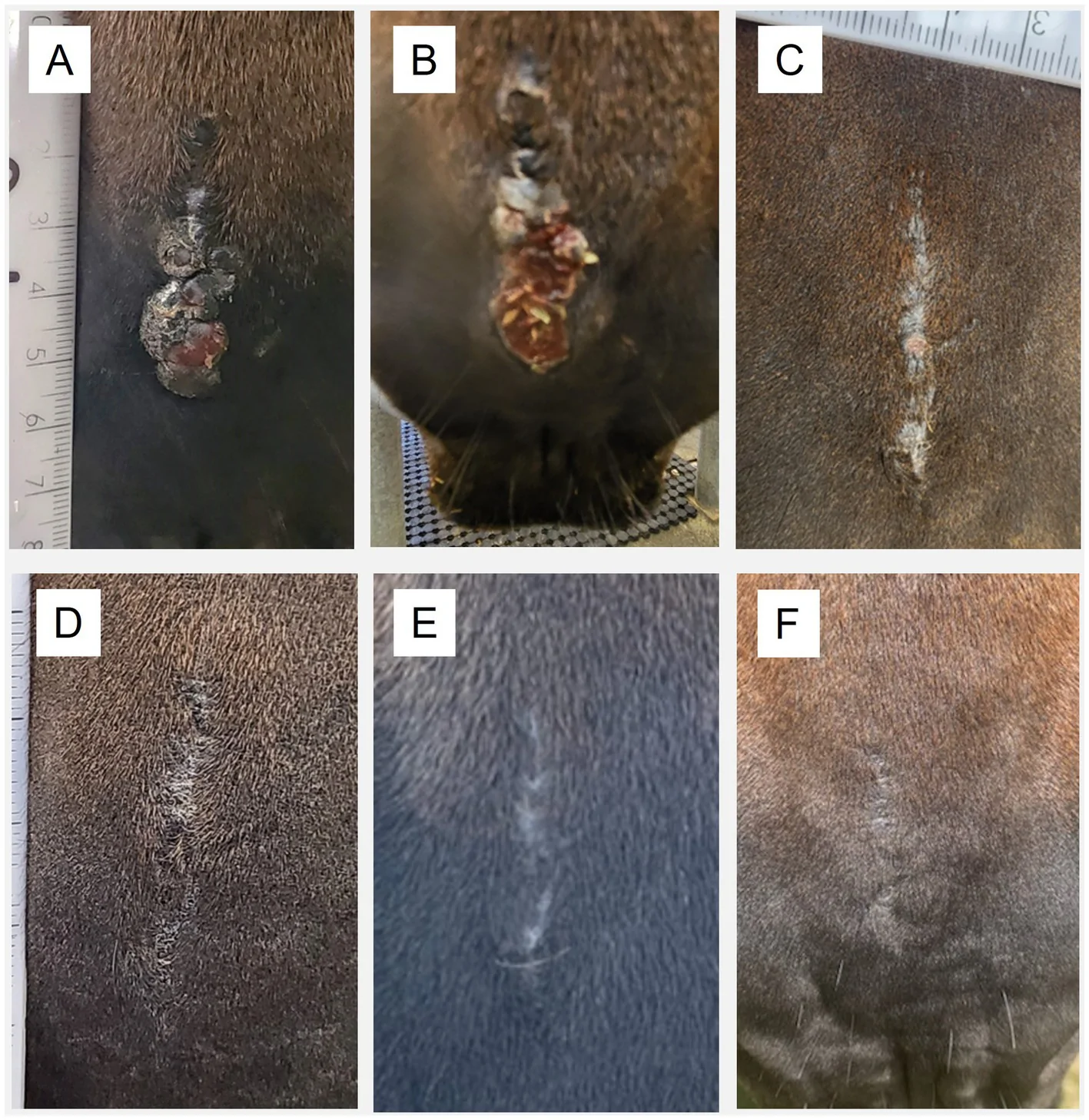
Clinical & temporal response to a single low dose treatment of a mixed verrucose/fibroblastic sarcoid (T_V_) is demonstrated (Horse 4). **(A)** Shows the sarcoid prior to treatment; **(B)** Complete tumour necrosis, eschar formation & slough D10; **(C,D,E)** Wound healing progression & re-epithelialisation at 5, 6, & 8 months; **(F)** Complete healing, minimal scar tissue & mild leukotrichia at 24 months.

The clinical and temporal response to a single low dose treatment of a mixed (verrucose/fibroblastic) is demonstrated in Figure 7. Figure 7A shows the tumour prior to treatment. Mild inflammatory signs and swelling was observed following treatment. During the treatment period the horse maintained a normal demeanour and healthy appetite. Complete tumour necrosis, eschar formation and slough occurred by D10 (Figure 7B). Rostrally the eschar had detached at this stage with evidence of healthy granulation tissue underneath filling the wound. Wound healing progressed uneventfully with re-epithelialisation occurring within ~3-weeks from treatment administration. Figures 7C–E demonstrate longer term follow-up (5,6 and 8 months respectively) with evidence of complete healing, minimal scar tissue formation and development of mild leukotrichia. Complete tumour resolution with no evidence of recurrence and minimal scar formation was observed on 24 months follow-up (Figure 7F).

### 3.3 Tigilanol tiglate loaded bead implantation

#### 3.3.1 T_VI_—right cranial pinna

**Figure 8:**
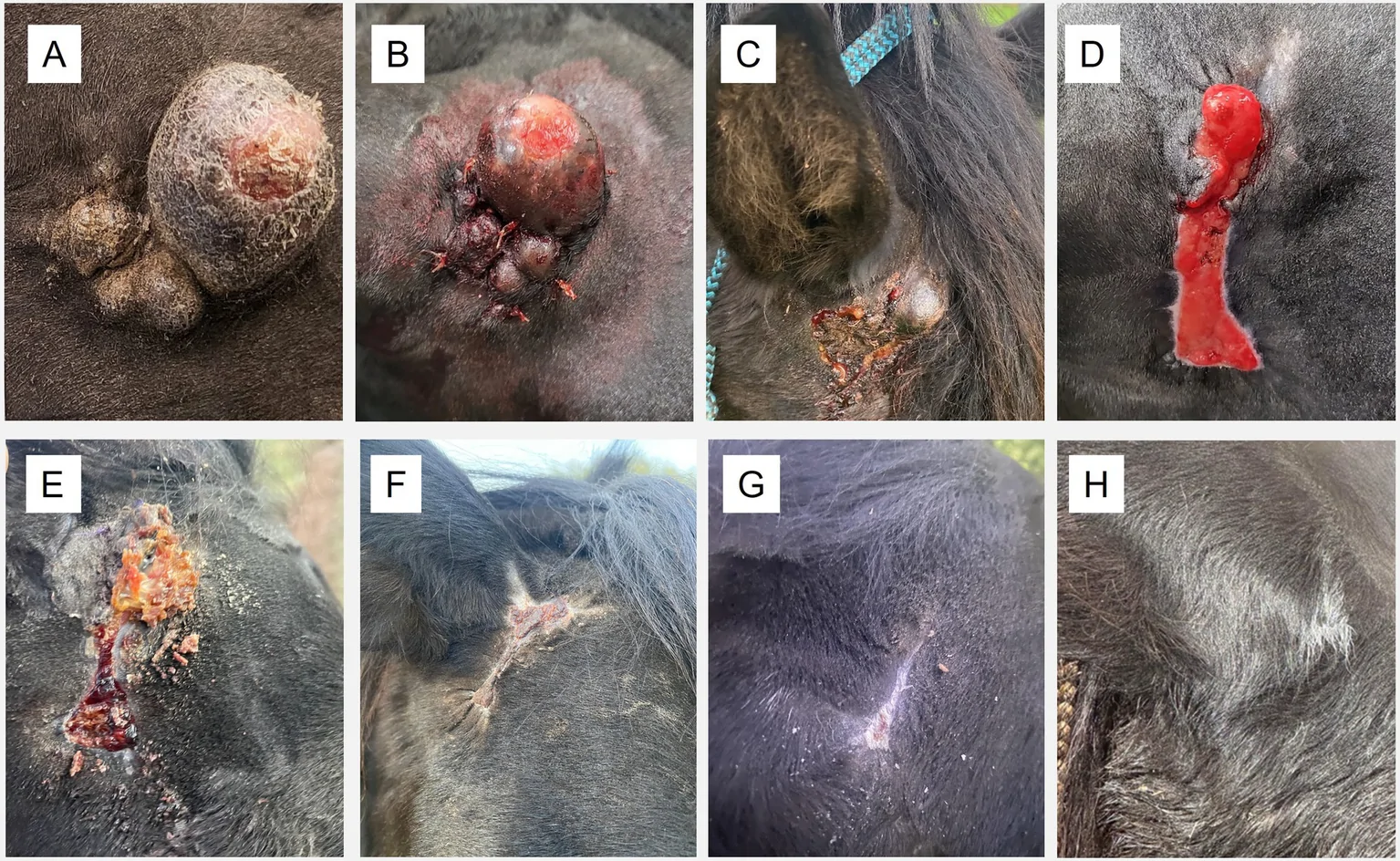
Clinical & temporal treatment response of a fibroblastic sarcoid (T_VI_) associated with the right pinna, is demonstrated. **(A)** Extent of sarcoid growth prior implantation of loaded beads; **(B)** Implanted beads & associated closed skin incisions; **(C)** Well demarcated area of necrosis D5; **(D)** Incomplete necrosis, sloughing and consequently incomplete wound healing by D21; **(E)** Exudative discharge & eschar formation D10 (T2); **(F)** Complete slough; **(G)** Full healing D45 (T2); **(H)** Complete tumour resolution with mild leukotrichia at 12-months.

The clinical and temporal treatment response of a fibroblastic sarcoid associated with the right pinna, is demonstrated in Figure 8. Figure 8A shows the extent of sarcoid growth prior to surgical implantation of tigilanol tiglate loaded beads. In Figure 8B beads have been implanted and associated skin incisions closed. Following mild swelling and oedema formation post bead implantation (D1-D2) a well demarcated area of necrosis was appreciated by D5 (Figure 8C). However, the more caudal part of the treatment site, involving a near spherical component of the fibroblastic sarcoid (tumour site A, volume = 4.95 cm^3^), demonstrated incomplete necrosis, sloughing and consequently incomplete wound healing by D21 (Figure 8D). A second bead treatment (T2) was performed and this time led to complete demarcation and necrosis of the sarcoid around D5 to D7 (T2), with development of exudative discharge, eschar formation (Figure 8E) and slough (Figure 8F). Full healing was observed at D45 post final treatment (T2; Figure 8G). 12-month follow-up confirmed complete tumour resolution with no evidence of recurrence at the treatment site (Figure 8H). During the treatment periods and following bead implantation the horse maintained a normal demeanour and appetite with no reported loss of pinna function or development of head shyness (resentment to head, ear or face being touched). Mild leukotrichia developed at the treatment site. No additional sarcoids developed elsewhere during the follow-up.

#### 3.3.2 T_VII_—right ventral muzzle

**Figure 9:**
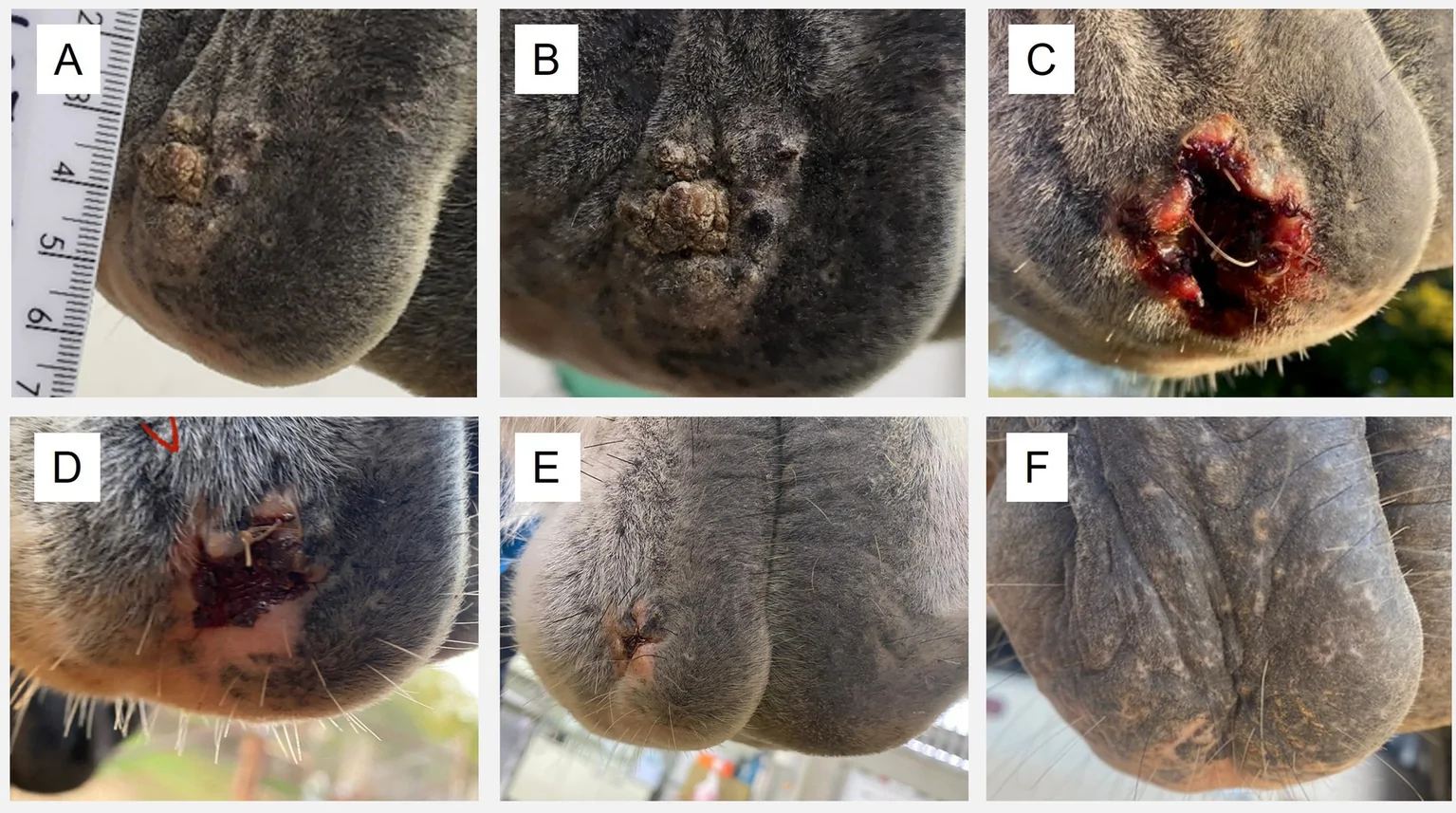
Temporal progression of the clinical treatment response of a verrucose sarcoid (T_VII_), associated with the right ventral muzzle, is demonstrated. **(A,B)** Demonstrates the sarcoid & its size prior to implantation of loaded beads; **(C)** Complete necrosis & tumour slough D7; **(D)** Granulation tissue filled deficit; **(E)** Healing by second intention, contraction D31; **(F)** Complete tumour resolution at 12-months.

Temporal progression of the clinical treatment response of a verrucose sarcoid, associated with the right ventral muzzle, is demonstrated in Figure 9. Figures 9A,B demonstrates the sarcoid and its size prior to surgical implantation of tigilanol tiglate loaded beads. As shown in Figure 9C complete haemorrhagic/avascular necrosis and tumour slough occurred by D7 following bead implantation. Minimal peritumoural inflammation and swelling was present during the post implantation period. The horse maintained a normal appetite and apprehended food normally throughout the treatment period. Granulation tissue filled the tissue deficit and the wound healed by second intention without incident (Figure 9D). Evidence of re-epithelialisation and wound contracture was observed around D28. No further treatments were administered and as demonstrated in Figure 9F, complete tumour resolution with no evidence of recurrence at the 12-month follow-up was observed. No additional sarcoids developed throughout the horse’s follow-up.

### 3.4 Elution of tigilanol tiglate from calcium sulphate beads

#### 3.4.1 5 mm beads (large)

**Figure 10:**
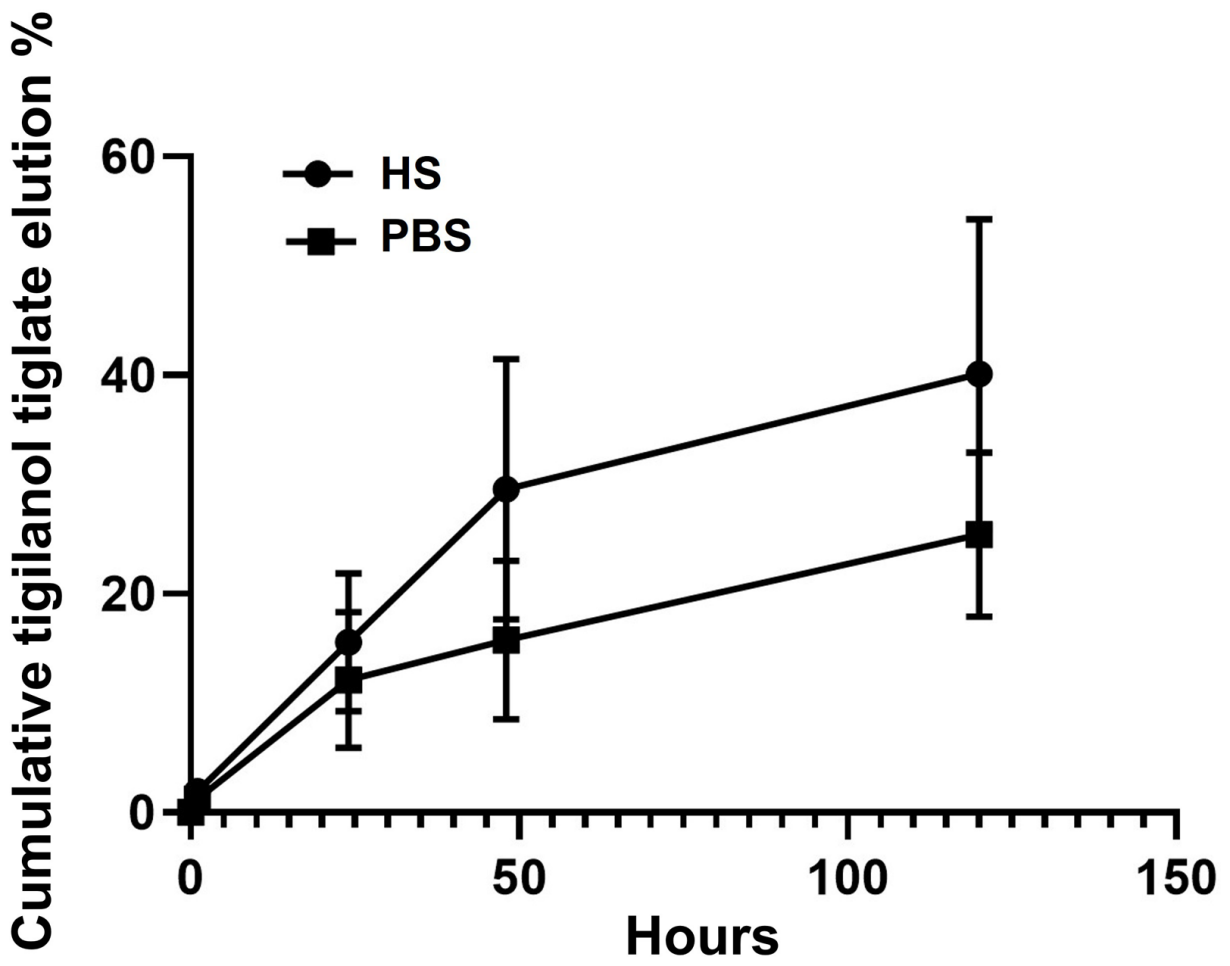
Cumulative dissolution curve of Tigilanol tiglate content from intact 5 mm absorbable calcium sulphate beads in horse serum and phosphate buffered saline over time. Error bars represent mean and standard deviation of triplicate analysis.

From the triplicate analyses, 5 mm tigilanol tiglate loaded beads had an average weight of 109.30 mg (SD = 1.46) and contained an average of 0.41 mg tigilanol tiglate (SD = 0.02). Assuming this average tigilanol tiglate content in all beads, analyses showed beads eluted ~40% of their tigilanol tiglate content in horse serum (HS) and ~26% in phosphate buffered saline (PBS; Figure 10). No tigilanol tiglate was detected in control beads. Rapid release of tigilanol tiglate from the 5 mm calcium sulphate beads occurred during the first 24 h, with continued release observed at 120 h from intact beads (Figure 10).

#### 3.4.2 2.5 mm beads (small)

**Figure 11:**
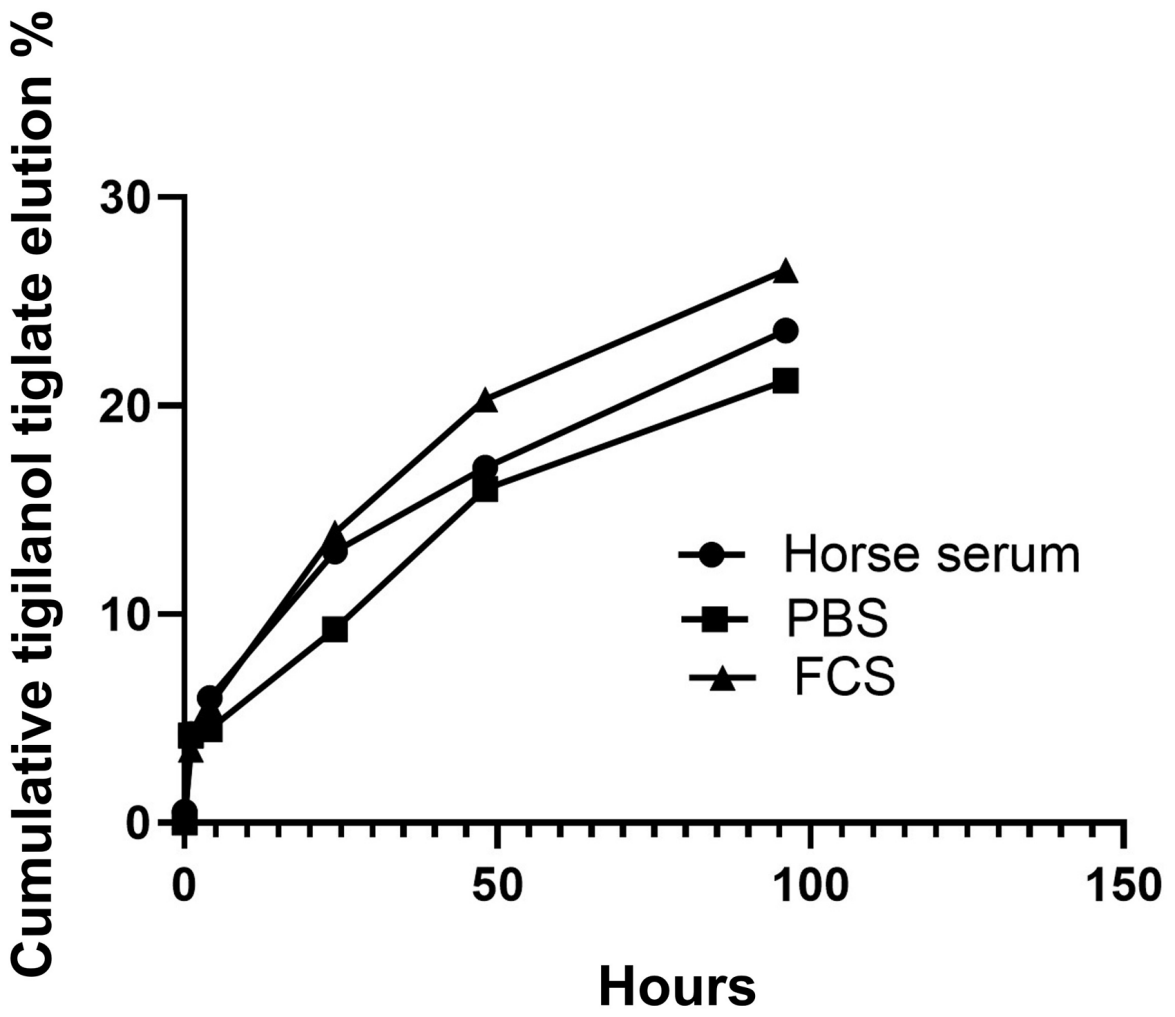
Cumulative dissolution curve of Tigilanol tiglate content from intact 2.5 mm absorbable calcium sulphate beads in horse serum, phosphate buffered saline and calf foetal serum over time.

2.5 mm Tigilanol tiglate loaded calcium sulphate beads had an average weight of 31.55 mg (SD = 0.62), with an average of 0.15 mg tigilanol tiglate (SD = 0.01) per bead. Assuming this average, between 21 and 26% (~24% HS, ~21% PBS, ~26% FCS) of available tigilanol tiglate was released from the calcium sulphate beads over a 2-day period (Figure 11).

Minimal formation of hydrolysed or rearranged forms of tigilanol tiglate was noted in both bead analyses.

## 4 Discussion

The case examples presented here have demonstrated an effective clinical response to substantially lower than previously reported treatment doses of tigilanol tiglate and provided proof of concept for this dose reduction in treatment approaches using either metronomic schedules, drug-eluting beads or a single intratumoural injection. Treatments resulted in complete tumour slough in all instances while subjectively also eliciting less pronounced local tissue responses when compared to previously established doses (0.35 mg tigilanol tiglate/cm^3^ tumour volume) (1, 2). In line with metronomic low dose intra-tumoural injections, elution data of the tigilanol tiglate loaded calcium sulphate beads established the potential for similar but delayed dose deliveries (rapid release in first 24 h but extended for 4 days) which were capable of inducing tumour necrosis and full resolution of disease.

Equine sarcoids remain one of the most prevalent cutaneous tumours encountered in horses (32–34). Although intralesional cytostatic drugs are an important treatment option for cutaneous neoplasia, intra-tumoural administration of naturally occurring and semi-synthetic epoxytiglianes (tigilanol tiglate) has demonstrated therapeutic efficacy causing rapid oncolysis and tumour slough with complete tumour regression in 73–74% of treated cutaneous tumours (1, 2). Intra-tumoural administration of tigilanol tiglate in horses has been associated with potential comorbidities (1, 2). An apparently lower inflammatory threshold compared to other species, was proposed to lead to more pronounced tissue responses at treatment sites (1, 2). Even so, this inflammatory response is integral to the multifactorial agnostic mode of action of tigilanol tiglate that leads to avascular oncolysis and recruitment of immune cells (2, 3, 35). Marked swelling, oedema and extensive tissue necrosis beyond treated tumour margins have been reported (1). Large defects occurring in sensitive and/or intricate anatomical sites such as the peri-ocular region, a commonly affected site, impose undesired management challenges (1). Unfavourable responses may be difficult to remedy despite instituting previously recommended equine-specific protocols. These include a 30% reduction in intratumoural dose rates compared to other species (canine and human) and provision of concurrent anti-inflammatory medications (1, 2, 33).

In accord with our hypotheses, we did not observe such complications by instituting both a low dose intra-tumoural injection of tigilanol tiglate or with eluting implants. Metronomic treatment regimens with cytostatic chemotherapeutic drugs have been utilised in human and veterinary medicine with the aim to reduce adverse effects, toxicity and lower the risk of potential drug resistance development (11, 12). Repeated delivery of tigilanol tiglate at lower doses may have reduced adverse effects in cases included in this study. A less pronounced inflammatory response was subjectively observed, judged by the minimal peri-tumoural swelling and oedema development post treatment. However, these observations were based solely on clinical assessment, without objective measurements. As such, conclusions regarding attenuation of inflammatory responses should be interpreted within this context. Though this lower dose treatment approach led to a perceived slower onset of tissue responses, time to complete tumour necrosis and slough was comparable to previous reports of cutaneous tumours treated with tigilanol tiglate (D6-10) (1).

None of the peri-ocular sarcoids treated with low doses of tigilanol tiglate in this study developed adverse tissue responses that affected eyelid function, caused ocular pain, or resulted in significant tissue defects. Consequently, this approach may suggest a lesser reliance on preemptive placement of sub-palpebral lavage (SPL) kits and administration of ocular medications as previously suggested (1). The peri-tumoural and distal limb swelling/oedema that developed following treatment of a sarcoid associated with the left-fore antebrachium (T_II_) was unexpected. However, given the anatomical location of the tumour and the self-limiting nature of the swelling in the absence of additional intervention, it may have been the result of an impaired lymphatic system.

The excellent response following treatment of a verrucous sarcoid (T_III_, T_IV_, T_V_ and T_VII_) is noteworthy and warrants further consideration. This type of sarcoid has previously proven particularly resistant to treatment (36, 37). Penetration of topically administered oncolytic drugs is limited by the thick keratinous layer associated with these sarcoids (37). Immunotherapeutic treatment, recently explored in the treatment of equine sarcoids, was also shown to be less effective in eliminating extensive verrucous sarcoids compared to single or multiple occult, nodular and fibroblastic sarcoids (36). Ineffective treatment followed by aggressive regrowth is common. This may be minimised by a widespread, homogenous distribution of oncolytic drugs (36). Multi-needle injectors have the ability to improve drug penetration and uniformity of distribution compared to single-needle administration (38). Delivery of tigilanol tiglate through a 34G multi-needle injector (Quatron®)^8^ was well-tolerated by horses. This method substantially improved the ease of administration of tigilanol tiglate when treating verrucose sarcoids. This combined with the metronomic administration regimen likely contributed to the effective treatment.

Despite the observed clinical efficacy from a single intratumoural injection, treatment of dense and fibrous tumours, particularly fibroblastic sarcoids, remains difficult. Reports of inadvertent seeding of tumour cells and treatment failure related to inadequate distribution of chemotherapeutic drugs highlight the need for alternative methods to optimise drug delivery, such as absorbable eluting beads (2, 27, 39). Methodology relating to the surgical implantation of loaded beads was adapted from reports treating sarcoids with biodegradable cisplatin beads (39). The ease of implanting absorbable tigilanol tiglate loaded calcium sulphate beads and its efficacy for the treatment of sarcoids was demonstrated. By utilising loaded beads, the potential risk of unsolicited diffusion from injection sites due to inherent difficulties of injecting fibrous tissue was eliminated. As judged by the restricted area of tissue necrosis (T_VI_ and T_VII_), loaded beads increased the perceived precision in dose administration. This contributed to the overall reduction in morbidity and observed downregulation of inflammatory responses in this study.

Peri-tumoural swelling in the two sarcoids (T_VI_ and T_VII_) treated with loaded calcium sulphate beads resolved within 48 h and without systemic administration of dexamethasone. This response was deemed mild compared to moderate swelling and redness lasting as long as 3 weeks following sarcoid treatment with cisplatin loaded calcium sulphate beads (39). One sarcoid (T_VI_) treated with tigilanol tiglate loaded calcium sulphate beads required a single repeat treatment. This was not unusual given the relatively large size of the tumour and the reported variable treatment response of sarcoids. Similarly, sarcoids treated with cisplatin loaded absorbable beads reportedly required on average two treatments while an average of four treatments was required when using an injectable formulation of the same chemotherapeutic drug (39).

Biodegradable loaded calcium sulphate beads created and utilised in this study consisted of synthetic calcium sulphate combined with tigilanol tiglate. 5 mm and 2.5 mm beads contained 0.41 mg and 0.15 mg tigilanol tiglate, respectively. This equates to a tigilanol tiglate content percentage of ~0.4% per bead irrespective of size. Content uniformity of prepared beads was not formally evaluated and therefore the consistency of tigilanol tiglate distribution among individual beads cannot be confirmed. Triplicate analysis of the large (5 mm) loaded calcium sulphate beads however showed the remaining tigilanol tiglate percentage to be reasonably close (SD = 3.68% for 5 mm beads eluted with HS), suggesting uniformity of tigilanol tiglate distribution throughout the manufactured beads.

The continued release of tigilanol tiglate following rapid release in the first 24 h suggests diffusion of the drug through the pellet matrix. Bead size has been shown to significantly affect *in vitro* elution from anti-microbial loaded calcium sulphate beads and has been ascribed to a difference in the surface area-to-volume ratio (18). Similarly, as demonstrated in Figures 10, 11, elution characteristics of the two loaded bead sizes prepared in this study showed the 5 mm beads (higher surface area-to-volume ratio) giving off a higher proportion of its tigilanol tiglate content compared to the 2.5 mm beads. As hypothesised elution occurred more rapidly in the 5 mm beads. Although the comparison between extraction solutions were not statistically evaluated, no discernible difference in dissolution profiles of tigilanol tiglate in HS, PBS and FCS were observed during assessment of the 2.5 mm beads. Based on this preliminary observation and the relevance of HS to clinical cases, only HS and PBS were used as extraction solutions for subsequent testing of the 5 mm beads. While aliquots (100 μL) were not replaced following sampling, cumulative release was calculated by correcting for the removed volume of extraction solution and the quantity of tigilanol tiglate removed in previous samples. The high protein binding affinity of the drug may have contributed to the observed profiles. Formation of hydrolysed or rearranged forms of tigilanol tiglate was negligible.

Though not a predefined study outcome, non-invasive thermographic assessment was performed on two treated lesions (T_I_ and T_II_) to evaluate changes in tissue perfusion and vascularity as a potential indicator of treatment response. Thermographic imaging has previously been shown to identify early vascular disruption and subsequent haemorrhagic necrosis in tigilanol tiglate treated canine mast cell tumours (3). Therefore, the localised reduction in surface temperature observed within treated sarcoid lesions (T_I_ and T_II_) were considered consistent with reduced perfusion and development of avascular necrosis.

While the oncolytic potential of low dose tigilanol tiglate for equine sarcoids was clear, observations are limited by the small study size, non-random administration of treatment and the variable follow-up time. Minimum effective treatment doses and tissue concentrations for tigilanol tiglate in equine sarcoids remain undetermined (40, 41). This work, however, illustrated that a single 0.05 mg/cm^3^ intratumoural dose (injection) of tigilanol tiglate, ~86% lower than the previously established (conventional) dose of 0.35 mg/cm^3^, is sufficient to achieve clinical tumour resolution. Likewise, a dose of 0.13 mg/cm^3^ (adjusted for bead elution), ~63% lower than the conventional dose, achieved clinical tumour resolution in a sarcoid treated with loaded calcium sulphate beads. Despite serial post-treatment tumour volume measurements not being performed, limiting quantitative assessment of tumour regression, the primary clinical outcome of complete tumour resolution remains unaffected.

This study did not assess potential systemic effects of tigilanol tiglate administered locally either by injection or loaded beads and given the lack of such effects reported to date is unlikely to be of relevance. The lack of complete independence between lesions (T_I_ and T_II_) treated concurrently within the same horse (horse 1), and the potential for systemic additive effects could have affected the broader interpretation of treatment responses in this horse. One horse (horse 1), contributing two sarcoid lesions (T_I_ and T_II_), was lost to follow-up before completion of the study due to poor owner compliance. Consequently, longer-term outcomes of these lesions could not be reported, representing a study limitation. However, the initial response of both lesions was consistent with expectations and the primary treatment outcome, i.e., complete tumour necrosis and sloughing. As such, the absence of long-term follow-up on these two lesions was unlikely to have materially affected our overall conclusions.

The aesthetic outcome of treatment sites in this study, irrespective of the regimen, was deemed excellent and comparable to that observed with use of higher doses of tigilanol tiglate. Complete tumour slough was achieved in all treated sarcoids. Wounds healed by second intention and resulted in minimal scar formation. Mild leukotrichia developed at three treatment sites which is a common sequel to hair-follicle or melanocyte damage induced by chemotherapeutic agents administered locally for treatment of skin tumours (28, 39, 42).

Further to the potential utility of our results in future applications, calcium sulphate has been proposed as a bone substitute in bone regeneration strategies (15–17, 43–45). Despite lacking intrinsic osteogenic or osteoinductive properties, its osteoconductive properties have proven it can act as a scaffold for new bone formation when applied to bone defects (17, 45). When combined with additives, bone resorption may be delayed and regeneration enhanced with the supplementary benefit of antimicrobial properties (46, 47). The antimicrobial activity of the epoxytiglianes and their effectiveness against important pathogens has previously been demonstrated (46, 48–50). Recently a semi-synthetic epoxytigliane (EBC-1013) demonstrated ability to modify bacterial growth and disrupt biofilm (46, 48–50). This epoxytigliane stimulates protein kinase C(PKC)-dependent neutrophil reactive oxygen species (ROS) and NETosis through local immune cell induction (46, 48–50). Concurrently, it lead to expression of cytokines, chemokines, and antimicrobial peptides in keratinocytes and fibroblasts,rapid resolution of infection and promotion of wound healing (46, 48–50). Having documented the loading and *ex vivo* elution characteristics of calcium sulphate beads containing tigilanol tiglate, the combined characteristics of calcium sulphate and an epoxytigliane may prove beneficial in various applications including treatment of bone neoplasia, infection or healing of bone defects (44, 47, 50).

Overall, the markedly lower doses of tigilanol tiglate, irrespective of the administration regimen, maintained sufficient bioactivity to achieve complete tumour resolution of treated sarcoids at perceived lower inflammatory tissue responses. Apart from the horse lost to follow up, treatment sites are still being monitored for long term follow-up with no evidence of regrowth at treated sites. Ongoing studies evaluating the clinical efficacy and safety of lowered tigilanol tiglate doses in a statistically relevant, representative equine population are required to refine the reported and proposed treatment regimens. The combined anti-cancer and potential bone regeneration properties of epoxytigliane loaded calcium sulphate beads together with their antibiofilm characteristics may warrant further exploration in oncological and orthopaedic applications.

## Data Availability

The raw data supporting the conclusions of this article will be made available by the authors, without undue reservation.

## Footnotes

1 Ceva Animal Health Pty Ltd., Glenorie, NSW, Australia.

2 ASTI Corporation, Tokyo, Japan.

3 Randlab Australia Pty Ltd., Chipping Norton, NSW, Australia.

4 Troy Laboratories Pty Ltd., Glendenning, NSW, Australia.

5 Norbrook Laboratories Australia Pty Ltd., Tullamarine, VIC, Australia.

6 Wright Medical technology Inc., Memphis, TN, United States.

7 Merck KGaA, Darmstadt, Germany.

8 Shimadzu Oceania Pty Ltd., Rydalmere, NSW, Australia.
